# Optimizing high mass flow parameters for high rate laser metal deposition of dense tungsten carbide iron composites

**DOI:** 10.1038/s41598-026-55884-0

**Published:** 2026-06-05

**Authors:** Haytham Elgazzar, Hassan Abdel-Sabour, Khalid Abdel-Ghany

**Affiliations:** The Advanced Digital Manufacturing Department, Central Metallurgical Research and Development Institute (CMRDI), ELflzzat, Tibbin, Cairo 11421 Egypt

**Keywords:** Additive manufacturing (AM), Laser metal deposition (LMD), Fe-based alloy, Tungsten carbide (WC), Composites, Industry 5.0, UN sustainable development goals (SDGs), Engineering, Materials science

## Abstract

Laser metal deposition (LMD) is a vital technology for the repair and surface protection of large-scale engineering components. Yet, its broader adoption within Industry 5.0 remains constrained by low deposition rates and the propensity for defects when high ceramic reinforcement fractions are employed. This study examined the LMD of an Fe-based self-fluxing alloy reinforced with 50 wt% WC at powder feed rates (*F*) of 45–140 g/min, laser energy densities (*E*) of 7.5–15 J/mm^2^, and interaction times (*T*) of 0.022–0.045 s. Systematic variation of scanning speed (*v*) and powder feed rate (*F*), supported by ANOVA, identified a stable processing window characterized by low energy density, short interaction time, and high *F*. Within this window, the dense powder stream produced a pronounced shielding effect that limited dilution to <10 %, suppressed excessive carbide dissolution, and stabilized the melt pool, yielding fully dense, near-defect-free deposits with uniformly distributed, largely intact spherical WC particles and only localized interfacial dissolution. At the optimum condition (*E* = 12 J/mm^2^, *F* = 90 g/min), matrix hardness reached 550 HV through dendritic refinement and secondary carbide precipitation, while build rate and melting efficiency were simultaneously maximized. These outcomes enable thick, high-integrity layers at industrially relevant productivity levels. This achievement directly advances circular manufacturing by enabling high-rate, near-defect-free remanufacturing and protective coating strategies that extend component service life, reduce virgin-material consumption, and lower embodied energy. The results thereby align LMD with Industry 5.0 human-centric production and contribute measurably to the UN Sustainable Development Goals through enhanced resource efficiency and sustainable materials use in the energy, automotive, and heavy-industry sectors.

## Introduction

Additive Manufacturing (AM) has received significant attention and has grown rapidly due to its distinct advantages over competing technologies, including design freedom and material flexibility. These advantages make it a vital technology for modern, smart factories that reflect the evolution of Industry 5.0 and help meet the UN Sustainable Development Goals (SDGs)^1–3^. Among the various AM processes, laser beam powder bed fusion (PBF-LB/M) and laser metal deposition (LMD) are widely used for building complex 3D engineering components, depositing protective layers to prevent corrosion, and repairing engineering components^3–5^ The SLM process is commonly used for fabricating complex small parts, while the LMD process is commonly used for fabricating large parts, performing repairs, and/or depositing protective layers to withstand harsh environments^6–10^. In this process, a high energy laser beam is used to fuse a selected material in the form of powder or wire with the desired properties onto a metal surface^4,10,11^. Moreover, LMD has received considerable interest from policymakers and industry stakeholders due to its profound impacts in many industrial sectors, such as energy, aerospace, and automotive, which is reflected in the establishment of recommendations and process standards to promote the widespread application of this technology. Recent research has emphasized overcoming key LMD process limitations, such as low deposition rates and lower productivity compared to conventional processes, which hinder its broader adoption in some industrial sectors^11–13^. Several strategies have been explored to improve the LMD process, aiming to increase the production rate and enhance process stability. Achieving a high deposition rate represents a significant advancement in LMD, enabling deposition rates exceeding 150 cm³/h through increased powder/wire feed rates and laser power^4,9,11^. This progress addresses productivity limitations in traditional LMD, where rates are typically limited to  60 cm³/h, by optimizing process parameters such as energy density and powder feed rate to mitigate defects such as porosity, cracking, and lack of fusion^4,10,11^. However, achieving a high deposition rate requires sophisticated design of the deposition head, tailored powder properties, and precise control of processing parameters, especially energy density, interaction time, and powder feed rate, to minimize dilution, maintain aspect ratios, and maximize build rates without introducing defects^11–13^. Therefore, optimizing these deposition process parameters may offer a more economical route, as it does not require expensive hardware upgrades. In this regard, an iron-based alloy reinforced with ceramic particles appears to be an economically viable and cost-effective material that can be used for repairing or providing surface protection for engineering components at relatively low cost^14,15^ due to its compatibility with steel substrates, and superior tribological properties^14–18^. These powder materials are primarily iron-based, which are inexpensive alloys alloyed with desirable elements such as Cr,Ni,B, and Si^13–15^ to provide excellent corrosion resistance and mechanical properties^13–15^. Reinforcing such alloys with ceramic particles, such as TiC, SiC, and WC, to form metal matrix composites (MMCs) makes them an ideal material for numerous applications due to their cost-effectiveness, good chemical and mechanical properties compared to more expensive alternatives such as Ni and Ti-based superalloys^19–22^. WC is employed as a reinforcing phase due to its high hardness, excellent wettability with iron-based alloys, and chemical stability^13–15^. The addition of WC significantly alters the microstructural evolution of the deposited layer, primarily by refining dendrites and thereby increasing microhardness^17–20^. During the solidification process in LMD, WC particles partially dissolve, enriching the molten pool with W and C atoms. This enrichment leads to the in-situ precipitation of secondary carbides. Studies have shown that as WC content increases, the microstructure evolves from primarily $\alpha$-Fe dendrites to a composite structure containing $\gamma$-(Fe, Ni),$\mathrm { M_{23} C_{6}, M_{6}C}$ and $\mathrm {Fe_{3} W_{3}C}$ carbides^13,17,18^. The dissolution of WC promotes solid solution strengthening and the formation of hard, skeleton-like grain boundaries, which effectively impede dislocation motion. Moreover, increasing WC content refines the grain size significantly; for example, varying WC content from 0 to 30 wt% has been shown to result in a transition from coarse columnar crystals to finer equiaxed dendrites^6–8,17^. Despite the benefits of adding WC as reinforcement ceramic particles, high concentrations of WC can compromise the structural integrity of the deposited layer due to residual stress accumulation, porosity formation, and microcracking^6–8,10,16^. The rapid heating and cooling rates inherent to the LMD process generate steep temperature gradients, leading to significant residual tensile stresses, particularly at the interface^6,10,11^. In addition, WC has a significantly different coefficient of thermal expansion compared to the Fe matrix, and excessive WC addition (e.g., >20–40 wt%) often leads to stress concentrations and brittle fractures^6,7,20^. Microcracks usually initiate at the interface between the hard, brittle WC phase and the softer Fe matrix^6,7^. Furthermore, molecular dynamics simulations have shown that interfacial behavior and fracture mechanisms in bimetallic systems are critically governed by thermal parameters, such as preheating and pouring temperatures, which influence atomic-scale diffusion kinetics and mechanical reliability^23^. Using high laser energy in combination with high concentrations of WC can exacerbate these defects in the layers^6^. Recent studies have successfully obtained near defect free layers through process optimization^6–8,16,17^ . Despite these advances, several critical research challenges remain with high powder feed rates, including melt pool instability, phase segregation, and defect formation under high laser energy^4,9–11^. Hence, the use of low energy density in LMD should be investigated to avoid or minimize defect formation in the deposited layers. Furthermore, the combination of high WC concentration and high feed rate needs to be investigated to fully demonstrate the capabilities of the LMD process. These critical research gaps limit the industrial scalability of the LMD process and its alignment with sustainable manufacturing models. In addition, there are limited research efforts that focus on maximizing high-rate mass productivity while maintaining structural integrity, despite LMD’s potential impact through repairing/remanufacturing components in the energy and automotive sectors^4,11^. This study addresses these gaps by investigating the LMD of an Fe-based alloy reinforced with $50~\mathrm {wt}\%$ WC at high powder feed rates (45–140 g/min) and low laser energy density in order to increase the production rate of the LMD process and produce dense, near defect free deposits. The relationships between laser power, scanning speed, interaction time, and powder feed rate were extensively investigated, aiming to evaluate the influence of energy density, interaction time, and powder feed rate on the deposition characteristics of Fe-WC composites, with a focus on geometric control (dilution and aspect ratio) and efficiency and productivity (build rate and melting efficiency). By optimizing these parameters, the study identifies a processing window that balances high volumetric build rates with metallurgical integrity, which is essential for industrial surface protection and repair applications, and directly supports SDGs through reduced material waste and extended component lifespans. Moreover, the findings of this study are expected to demonstrate to policymakers and industry stakeholders the benefits of the LMD process, thereby helping to facilitate the development of regulations and attract more funding to promote its widespread adoption across diverse applications. This is particularly relevant as LMD can readily meet the standards and requirements of Industry 5.0 and the SDGs for sustainable and efficient manufacturing, which is highly recommended, especially for developing countries. Finally, in this study, a techno-economic analysis was deliberately omitted to maintain a focus on the relationships that govern the LMD of WC-reinforced Fe-based composites. These relationships, derived from controlled variations in energy density, interaction time, and powder feed rate, yield universally applicable insights into defect mitigation, carbide retention, and deposition efficiency that transcend specific geographic or industrial contexts. Economic feasibility assessments, by contrast, are inherently context-dependent, requiring localized input data on variables such as energy tariffs, labor rates, equipment amortization schedules, and supply chain dynamics factors that are best addressed through dedicated life-cycle assessment or techno-economic modeling frameworks once the underlying metallurgical reliability has been established. By prioritizing the identification of a stable processing window that delivers dense, high-integrity deposits at elevated productivity, the present work provides the essential process reliability foundation necessary to inform and validate subsequent region-specific or application-specific economic evaluations, thereby accelerating the pathway from laboratory optimization to industrial implementation.

## Experimental details

**Fig. 1:**
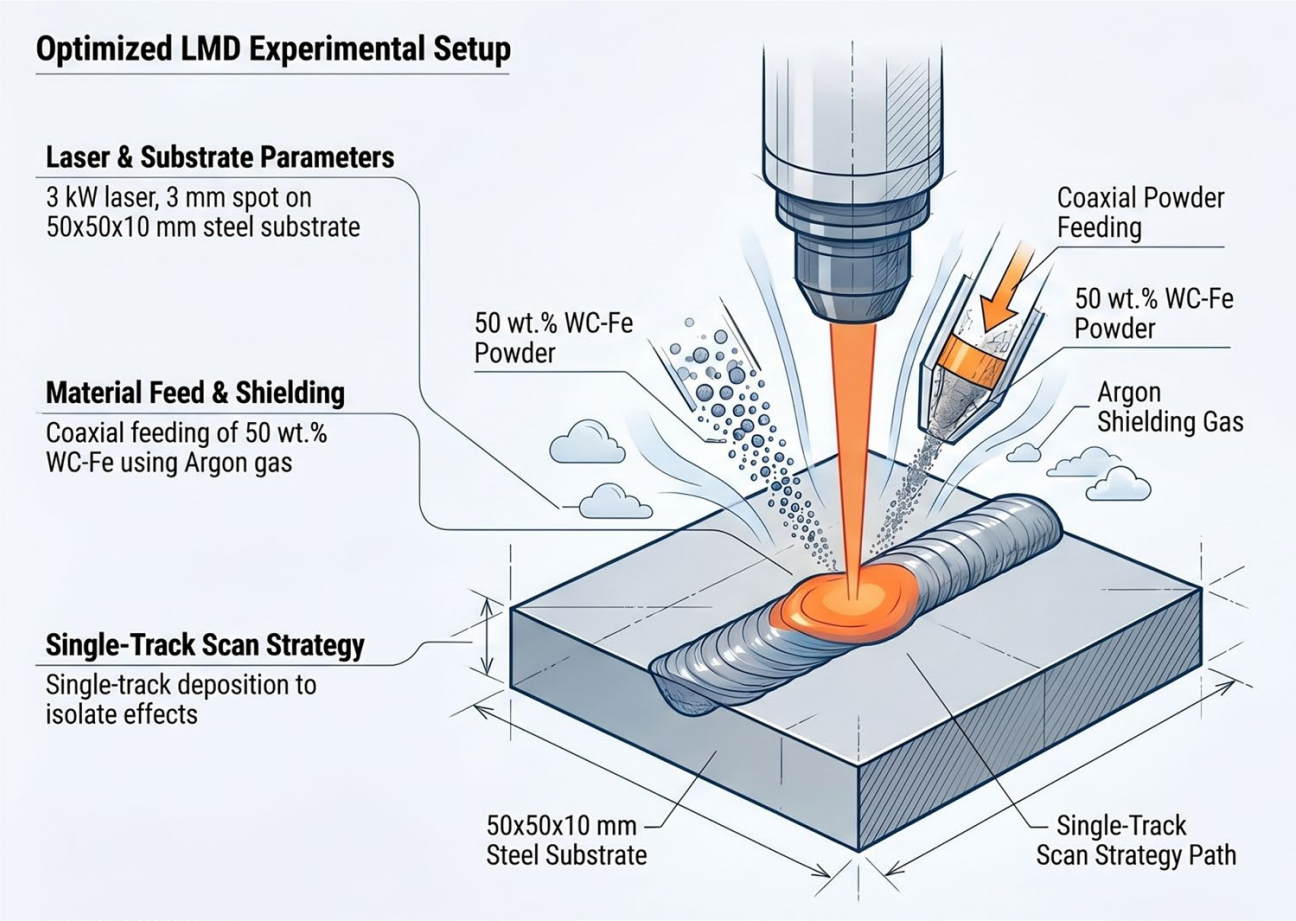
Schematic diagram of the coaxial laser metal deposition (LMD) process.

The commercial steel plates with dimensions $50\text {~mm} \times 50\text {~mm} \times 10\text {~mm}$ were selected as the substrate material. A pre-mixed powder consisting of an iron-based alloy with a size distribution of 60–150 $\mu$m and tungsten carbide (WC) with a size distribution of 50–105 $\mu$m in a 50:50 weight ratio was used as the deposition material. The chemical compositions of both the substrate and powder materials are listed in Table 1. Chromium and nickel provide excellent corrosion resistance. Boron and silicon act as self-fluxing agents, lowering the melting point and improving the fluidity of the melt pool during the LMD process and resulting in a dense, pore-free coating. The powder mixture was dried in a furnace at 90$^{\circ }$C for 4 h to remove moisture and then agitated using a mechanical agitator to ensure homogeneity. The LMD process was carried out using a 3 kW laser with a spot size of 3 mm. Powder feeding was performed coaxially by means of a powder feeder. A systematic experimental design was employed to investigate the effects of energy density, interaction time, and powder feed rate on the quality of the deposited layer (dilution, aspect ratio, build rate, and defects such as porosity and microcracks). Argon gas was used as both the powder carrier gas and the shielding gas to protect the coating against oxidation. The gas flow rates for all experiments were set to 5 L/min. The selected process parameter boundaries (scanning speed: 4000–8000 mm/min; powder feed rate: 45–140 g/min) were determined through a pre-experimental screening phase. Single-track deposits were produced across a wide range of parameters to identify stable processing conditions. A schematic illustration of the LMD process setup is shown in Fig. 1.

The upper scanning speed limit was defined by the onset of melt-pool discontinuity, while the lower limit was defined by excessive substrate dilution. The powder feed rate boundaries were set by the maximum and minimum stable feed rates achievable by the powder feeder, thereby ensuring consistent powder flow and complete melting of the majority of particles. The parameters used in the experiments are shown in Table 2. The experiments were conducted at room temperature without substrate preheating to isolate the effects of the shielding mechanism and the low energy density on crack mitigation. Substrate preheating was deliberately omitted in the present experiments to isolate the shielding effect of the dense powder stream on thermal gradients. The absence of macro-cracking despite the high (50 wt%) WC content demonstrates that the low-energy, high-feed-rate window effectively managed residual stresses; the influence of substrate preheating on thicker multi-layer builds will be examined in future work.

**Table 1:** The chemical composition $\%$ of deposited materials and substrates.

Material/Composition	C	Si	Cr	Ni	B	W	Other	Fe
Fe powder	0.079	1.36	16.49	5.31	1.8	0.011	0.0	Bal.
WC powder	3.8	–	–	–	–	Bal.	<0.7	–
Steel substrate	0.11	0.16	0.03	0.01	0.0	0.0	1.35	Bal.

The LMD parameters can be expressed by laser energy density [*E*], which is the applied energy per unit area, and can be calculated using Eq. 1^4,10^:

1 $$\begin{aligned} E = \frac{P}{d \cdot v} \quad [\text {J/mm}^2] \end{aligned}$$

where *P* is the laser power [W], *d* is the spot size [mm], and *v* is the scanning speed [mm/s].Energy density (*E*) was varied to investigate its effect on the melt pool and to determine the amount of energy needed to melt the steel substrate without causing vaporization or excessive dilution. The duration of the laser beam over a specific point is represented by interaction time, a further crucial parameter in the LMD process. Since the spot size (*d* = 3 mm) is fixed in this study, the interaction time is solely a function of *v*. The interaction time (T) is calculated using Eq. 2^4,12^:

2 $$\begin{aligned} T= \frac{d}{\ v} \quad [\text {s}] \end{aligned}$$

Given that laser power and spot size were held constant, both *E* and *T* are uniquely determined by and perfectly collinear with *v*. Consequently, the statistical analysis was performed using two independent factors: powder feed rate (*F*) and *v*. This approach maintains statistical validity in the ANOVA while accurately reflecting the physical coupling between *E* and *T*. ANOVA was used to quantify which parameters significantly affect the quality of the deposited layers (dilution, aspect ratio, build rate, and melting efficiency). To determine the quality of the deposited layers, the following indicators are measured or calculated:

1. The Dilution ratio (*D*), which is defined as the ratio of the melted area of the substrate to the total melted area. The dilution(*D*) was calculated by the Eq. 3^12^
  3 $$\begin{aligned} D = \frac{A_s}{A_s + A_c} \end{aligned}$$
  where $A_{s}$ is the melted area of the substrate, and $A_{c}$ is the area of the deposited layer.
2. Aspect ratio (*A*), which is defined as the ratio of bead width to height of the deposited bead and serves as an indicator of bead stability. The aspect ratio (*A*) was calculated by the Eq. 4^24^
  4 $$\begin{aligned} A = \frac{w}{\ h} \quad \end{aligned}$$
  where *w* is the width and *h* is the height of a single bead [mm]
3. Build rate (*B*), which is a primary indicator of the LMD process productivity and is defined as the volume of material deposited per unit time. The build rate (*B*) was calculated by the Eq. 5
  5 $$\begin{aligned} B = (w * h * v)\quad [mm^3/min] \end{aligned}$$
4. Powder melting efficiency (*M*): calculated as the ratio of the volume of material successfully deposited per unit of energy delivered. The melting efficiency (*M*)was calculated by the Eq. 6
  6 $$\begin{aligned} M = \frac{M_{c}}{\ M_{t}} [\%] \end{aligned}$$
  where $M_{c}$ is the mass of the deposited layer, defined as the actual mass of the deposited layer (mass after deposition minus mass before). $M_{t}$ is the total powder fed, defined as the total amount of powder delivered by the feeder during the process (powder feed rate x deposition time). To ensure reproducibility and consistency of results, each condition was tested in triplicate. For each replicate, single beads were deposited, and their dimensions (width and height) were measured using an optical microscope. The cross-sectional microstructures of the resulting layers were examined by scanning electron microscopy. For microstructural observation, all specimens were cut transversally, ground, polished with diamond paste, and etched with a reagent (100 ml $H_{2}$O +10g KOH+ 10g $K_{3}$[Fe($CN_{6}$)]). Microhardness along the depth of the cross-section of the layer was measured using a microhardness tester. The applied load was HV 0.3 and the loading time was set at 10 s.

**Table 2:** The main LMD processing parameters.

Power (W)	Powder feed rate (g/min)	Spot size (mm)	Gas flow rate (L/min)	Scan speed (mm/min)	Energy density (J/mm^2^)	Interaction time (s)
3000	45, 90, 140	3	5	4000	15.0	0.045
				5000	12.0	0.036
				6000	10.0	0.030
				7000	8.5	0.026
				8000	7.5	0.022

## Results and discussion

### Statistical analysis results

ANOVA was performed to quantify the impact of scanning speed (*v*) and powder feed rate (*F*) on the geometric and efficiency indicators, primarily dilution (*D*), aspect ratio (*A*), build rate (*B*), and melting efficiency (*M*). The results, presented in Table 3, show the relative contributions of *v* and *F*.

**Table 3:** ANOVA table for various responses.

Response	Source	F-value	*p* value	Contribution (%)
Dilution (D)	v	0.5	0.7404	2.03
	F	43.73	< 0.0001	89.76
	Residual error	–	–	8.21
Aspect ratio (A)	v	36.56	< 0.0001	40.14
	F	105	< 0.0001	57.66
	Residual error	–	–	2.2
Build rate (B)	v	5.34	0.0215	45.92
	F	8.59	0.0102	36.9
	Residual error	–	–	17.18
Melting efficiency (M)	v	13.31	0.0013	77.42
	F	3.77	0.0704	10.95
	Residual error	–	–	11.63

**Fig. 2:**
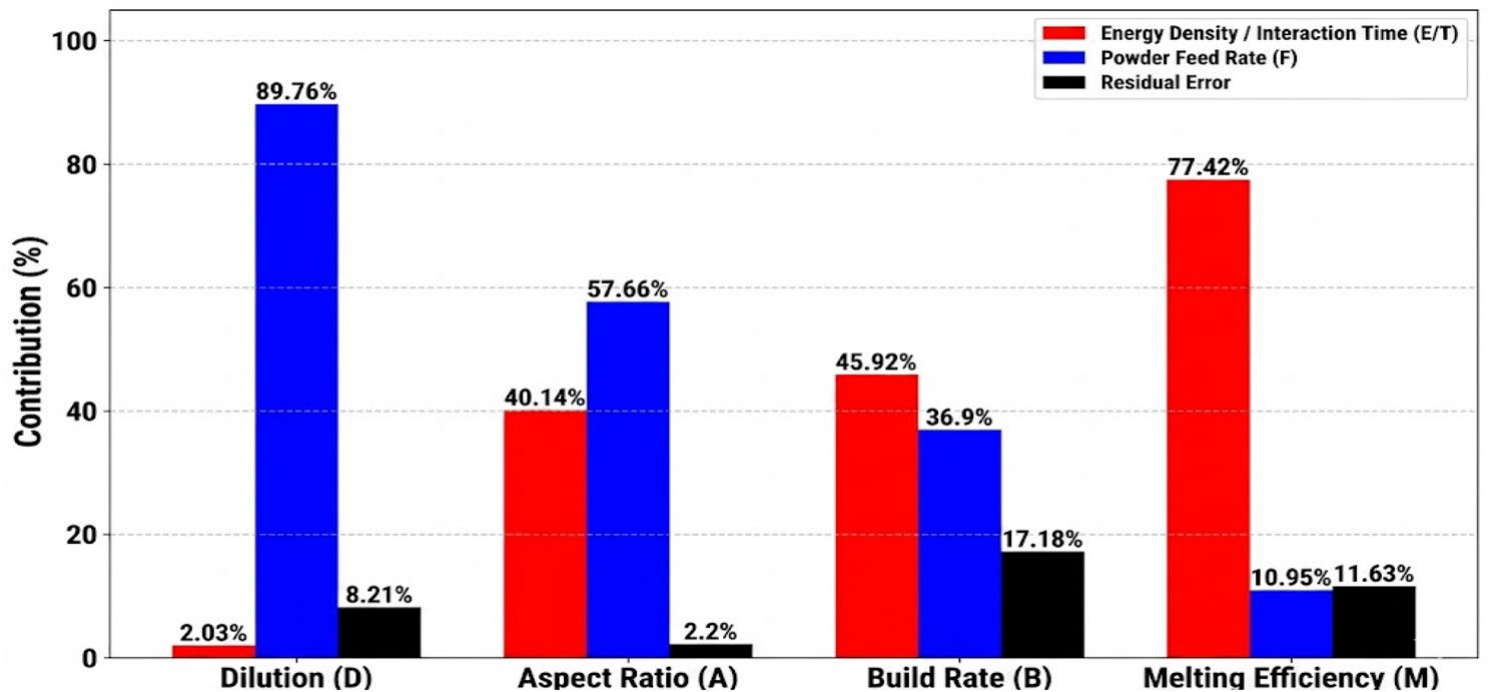
ANOVA statistical contribution of the LMD process parameters.

As shown in Table 3 and illustrated in Fig. 2, the governing mechanisms for geometric and efficiency characteristics vary significantly between powder mass flow-driven and energy-driven regimes. The ANOVA results showed that for geometric control (*D* and *A*), *F* is the dominant factor, contributing 89.76 $\%$ and 57.66 $\%$, respectively. This suggests that the physical volume of the delivered powder and the resulting shadowing effect on the incident laser beam are the main controllers of bead shape and substrate melting. In contrast, for process efficiency and productivity (*B* and *M*), *v* is the dominant factor, contributing 77.42$\%$ and 45.92$\%$, respectively. This indicates that while material is available, the system’s ability to process that material is primarily energy limited. The residual error for build rate (17.18 $\%$) is the highest among the responses, indicating some degree of uncontrolled variability. This is likely attributable to minor fluctuations in powder flow rate and the inherent instability of the melt pool at the extremes of the process window, which affect bead dimension measurements. Nevertheless, the high F-values for the main factors (*p* < 0.05) confirm that their influence on build rate remains statistically significant.

To verify the statistical adequacy and predictive accuracy of the generated ANOVA models, model validation parameters were systematically evaluated, as summarized in Table 4. The coefficient of determination ($R^2$) for the geometric responses, dilution (*D*) and aspect ratio (*A*), reached 91.79% and 97.80%, respectively, indicating that the selected independent parameters capture nearly all of the observed experimental variance. For the process productivity and efficiency indicators build rate (*B*) and powder melting efficiency (*M*) the models demonstrated robust fits with $R^2$ values of 82.82% and 88.37%, respectively. The close agreement between the adjusted $R^2$ ($\text {Adj.-}R^2$) and predicted $R^2$ ($\text {Pred.-}R^2$) across all models confirms the robust predictive capacity of the framework and the absence of overfitting. Furthermore, residual analysis was performed to validate the underlying ANOVA assumptions. Normal probability plots of the residuals revealed a strictly linear distribution, confirming that the experimental errors are normally and independently distributed with uniform variance (homoscedasticity). Due to the physical decoupling between the coaxial pneumatic powder feeder (*F*) and the robotic translation system (*v*), interaction effects between the two primary parameters were found to be statistically non-significant and were omitted to preserve the statistical degrees of freedom of the error terms. This low experimental scatter underpins the high precision and tight reproducibility of the triplicate runs.

**Table 4:** Model validation and fit summary parameters for the LMD experimental responses.

Experimental response	*R*^2^ (%)	Adjusted *R*^2^ (%)	Predicted *R*^2^ (%)	Model adequacy
Dilution (*D*)	91.79	85.63	71.18	Robust
Aspect Ratio (*A*)	97.80	96.15	92.27	High precision
Build Rate (*B*)	82.82	69.94	41.52	Satisfactory
Melting Efficiency (*M*)	88.37	79.65	59.81	Robust

### Influence of process parameters on bead geometry

#### Dilution control

**Fig. 3:**
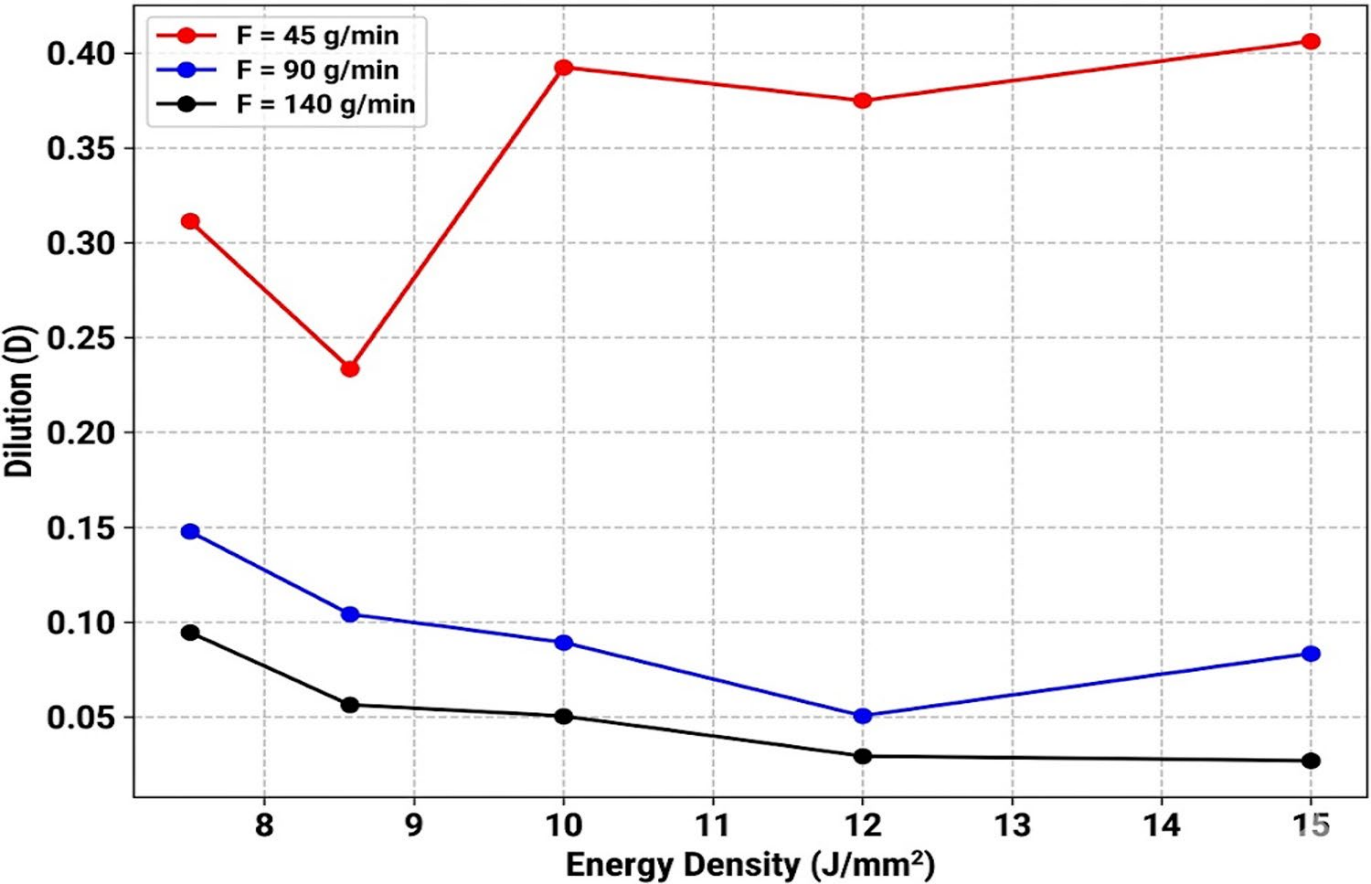
Effect of energy density on dilution.

The interaction between *v* and *F* significantly influences the stability of the processing window. As shown in Fig. 3, dilution (*D*) is primarily controlled by and inversely proportional to *F*, a relationship confirmed by the ANOVA results. *D* remains consistently low at higher *F* values, aligning with reported results in^9^. At *F* = 140 g/min, *D* remains below 10 % across the entire examined range of *E*. This is attributed to a powder induced shielding effect, where the dense particle stream attenuates the laser beam before it reaches the substrate. By preferentially absorbing laser energy for powder melting, this particle cloud acts as a thermal buffer that reduces the heat flux reaching the substrate, successfully lowering *D* from approximately 40 % to $<5$ %. While direct laser transmission measurements were not performed, the strong inverse correlation between *F* and *D* at constant laser energy provides compelling indirect support for this mechanism. Maintaining low *D* is critical for preserving the chemical integrity of the alloy while ensuring sufficient metallurgical bonding. Consequently, for precise control of *D*, optimizing *F* is more critical than adjusting *v* or laser power.

#### Aspect ratio

**Fig. 4:**
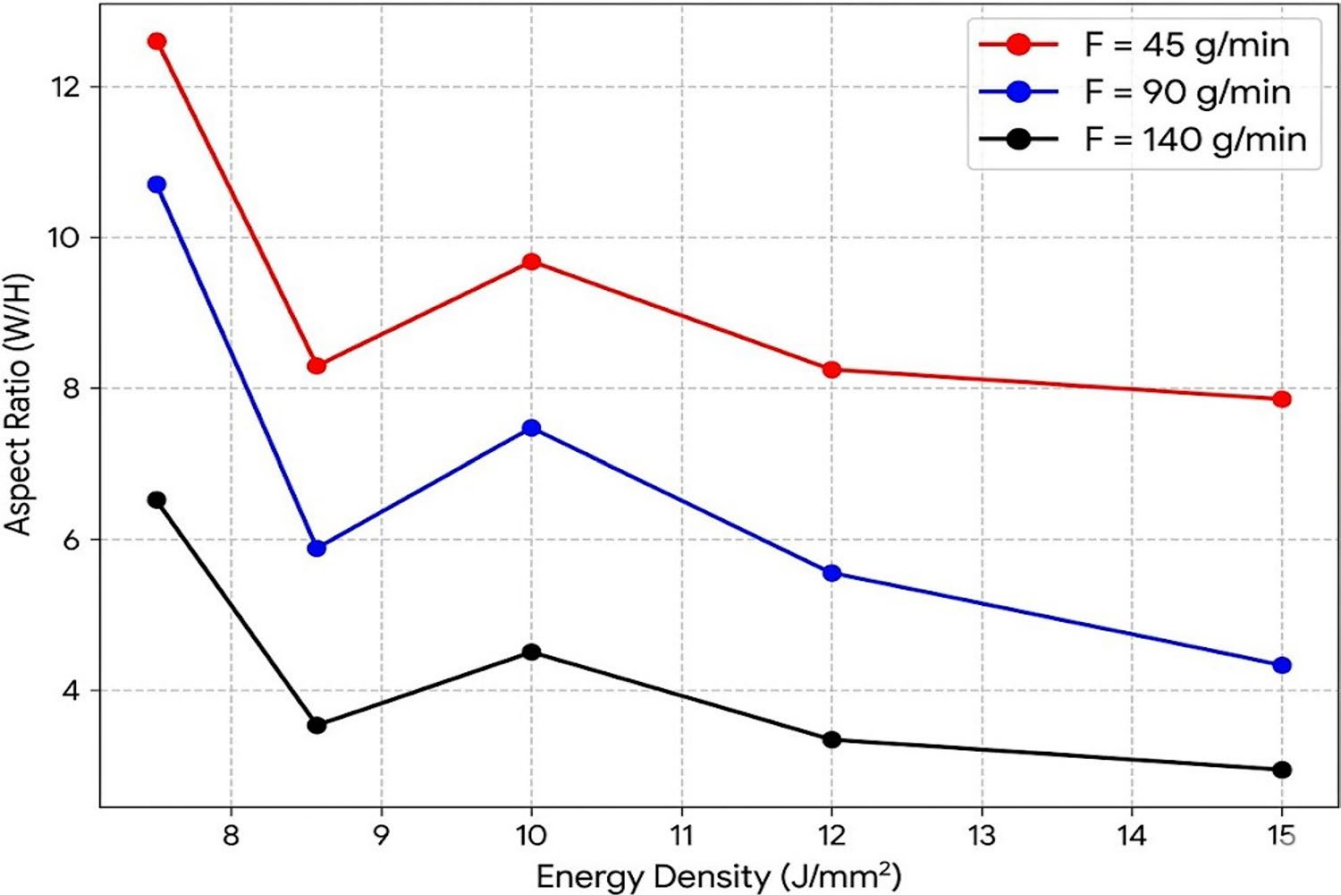
Effect of energy density on aspect ratio.

Figure 4 shows that the aspect ratio (*A*) decreases as both *v* and *F* increase, resulting in taller and narrower beads. This trend is consistent with the 57.66 % contribution of *F* to the variance in *A*, as shown in Table 3. The combined influence of both factors indicates that bead geometry is sensitive to the melt pool size (governed by *v*) and the volume of deposited material (governed by *F*). Moreover, the 40.14 % contribution of *v* to*A* (Table 3) is linked to the self-fluxing nature of the matrix powder, which contains silicon (1.36 wt%) and boron (1.34 wt%) as listed in Table 1. These elements reduce the surface tension of the melt pool, thereby enhancing fluidity and promoting bead spreading. In addition, WC particles require sufficient energy input to maintain a fluid melt pool that facilitates spreading of the molten material across the substrate and produces a wider bead.

### Process productivity and efficiency

#### Build rate dynamics

**Fig. 5:**
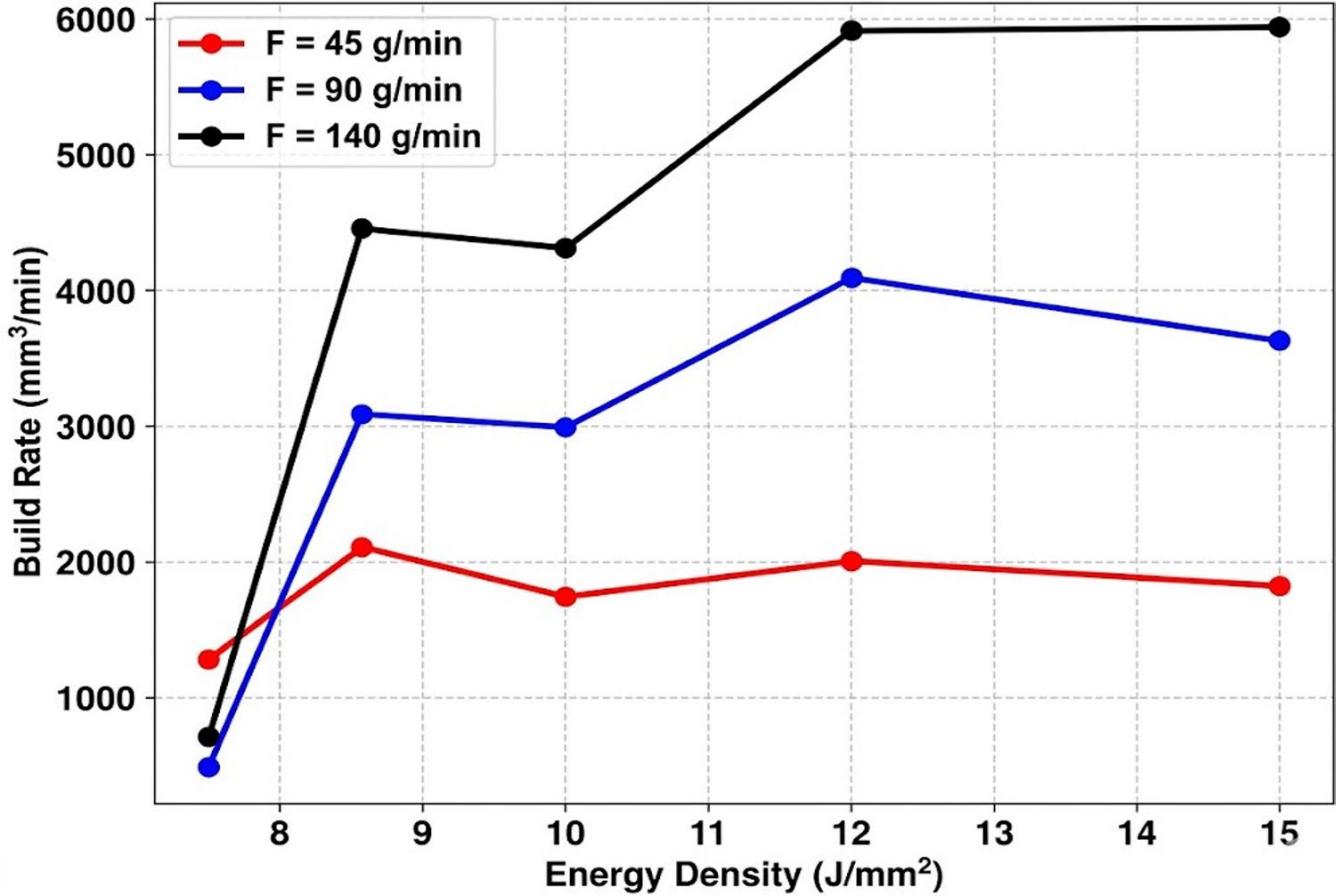
Effect of energy density on building rate.

The build rates (*B*) reported in the present work (Eq. 5) represent the volumetric productivity potential derived from single bead geometries. Multi layer wall or block deposition experiments are required to validate these rates under sustained thermal accumulation conditions and are planned for future work. These single bead metrics provide a useful comparative baseline for process efficiency. The influence of *v* and *F* on *B* is clearly demonstrated in Fig. 5. *B* shows a strong positive correlation with both *v* and *F*. However, as shown in Table 3, the higher statistical contribution of *v* (45.92 %) over *F* (36.9 %) indicates that increasing the mass flow is insufficient without a proportional increase in thermal input to effectively incorporate the powder into the build. This is particularly relevant for the WC powder, whose high carbon content (3.8 wt%), high melting point ( 2870 °C), and associated high latent heat of fusion ( 330–560 kJ/kg) require substantially higher thermal input for complete or partial melting and incorporation.

#### Powder melting efficiency (PME) and coating thickness

**Fig. 6:**
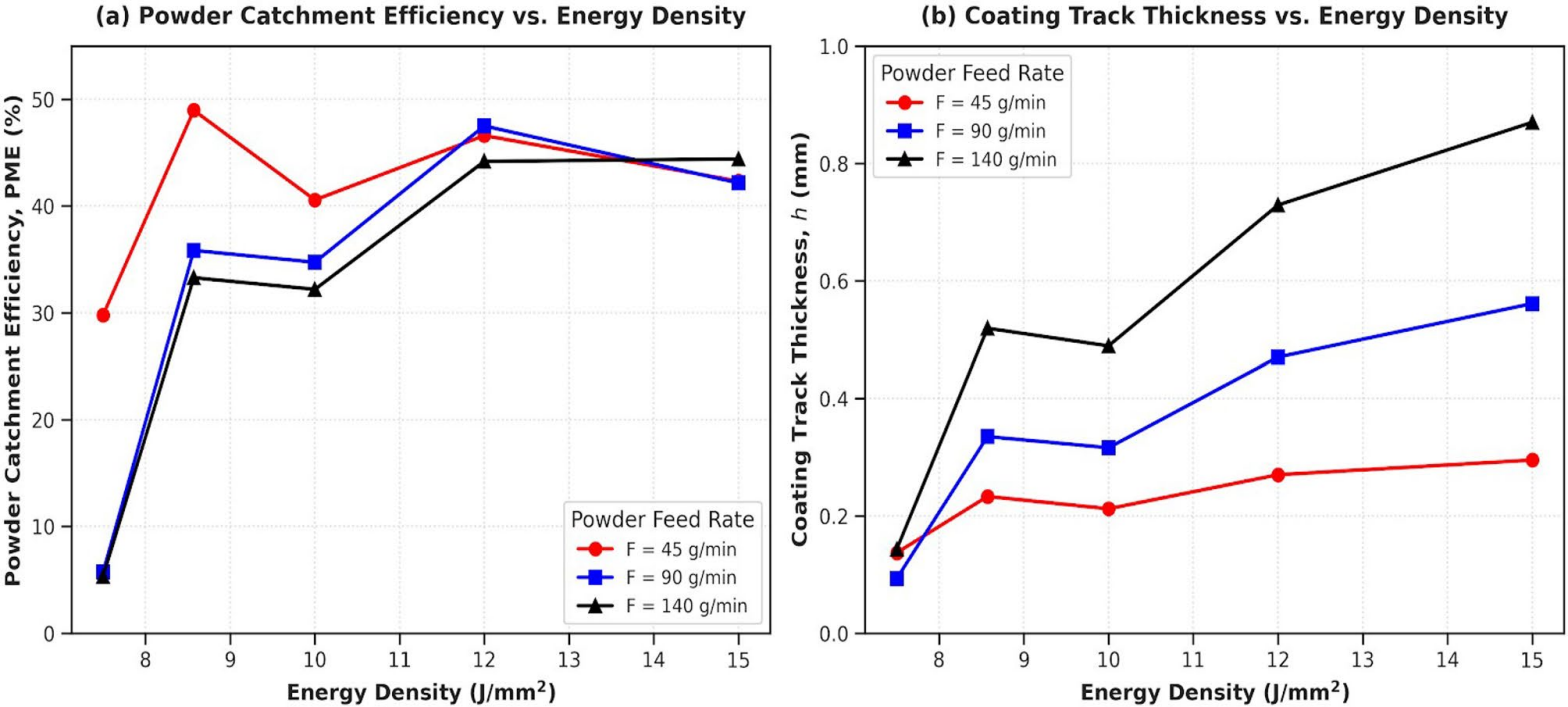
Evolution of deposition characteristics as a function of *E* across different *F*: (**a**) PME, %, (**b**) coating thickness (h, mm).

To establish the industrial scalability and material deposit economy of high-rate LMD for the 50 wt% WC-Fe composite, the Powder Melting Efficiency (PME) which serves as the operational measure of powder catchment efficiency must be evaluated in tandem with the final single-track coating thickness (bead height, *h*). The simultaneous evolution of these two critical process indicators across all parameter variations is illustrated in Fig. 6 as a function of energy density (*E*).

As displayed in Fig. 6, the process catchment behavior drops into distinct performance regimes determined by the volumetric energy input. Under a low powder delivery rate ($F = 45$ g/min), the PME values remain stable and highly efficient, ranging between 40.6% and 49.0% across a broad energy band, before dropping to 29.8% at the lowest energy density boundary ($E = 7.5\text { J/mm}^2$). Crucially, when processing heavier powder loads ($F= 90$ g/min and $F= 140$ g/min), the system undergoes a sudden process degradation at ($E = 7.5\;{\mathrm{J/mm}}^{2}$), where the PME collapses entirely to 5.7% and 5.3%, respectively. This collapse visually confirms the severe influence of the powder-induced shielding effect. At this low *E*, the ultra-short interaction time ($T = 0.022$ s) combined with a high density spatial cloud of un-melted carbide and matrix particles heavily scatters and attenuates the incoming laser beam. As a consequence, the net heat flux reaching the substrate surface is insufficient to sustain a stable melt pool, causing the majority of the delivered powder stream to deflect off cold rather than catch. Stabilizing the PME above 42% requires an *E* threshold of $12\text { J/mm}^2$ to successfully melt and incorporate these heavy mass streams. Parallel tracking of the resulting coating thickness (*h*) in Fig.6 demonstrates a highly predictable, proportional relationship with the laser energy density. At the maximum energy input of $15\text { J/mm}^2$, track thickness values peak at 0.29 mm ($F = 45$ g/min), 0.56 mm ($F = 90$ g/min), and a prominent 0.87 mm under the maximum high productivity delivery rate ($F = 140$ g/min). As the *E* decays, the track height undergoes a continuous, uniform drop due to the reduced linear mass deposition per unit area. These combined trends highlight that optimizing high-rate LMD coatings requires selecting a narrow operating window ($E \ge 12\text { J/mm}^2$) where both high powder melting efficiency and robust layer dimensions can be simultaneously maintained without causing process failure. A significant reduction in *M* is observed at the lowest *E* (7.5 J/$mm^2$), particularly at the highest *F* (140 g/min), where efficiency drops to approximately 5$\%$ due to partial melting of larger particles when the interaction time is insufficient. This results in substantial powder waste. Consequently, *v* must be adjusted proportionally with *F* to establish a stable melt pool and ensure that the latent heat of fusion is adequately supplied for the entire powder volume.

### Microstructure characterization

**Fig. 7:**
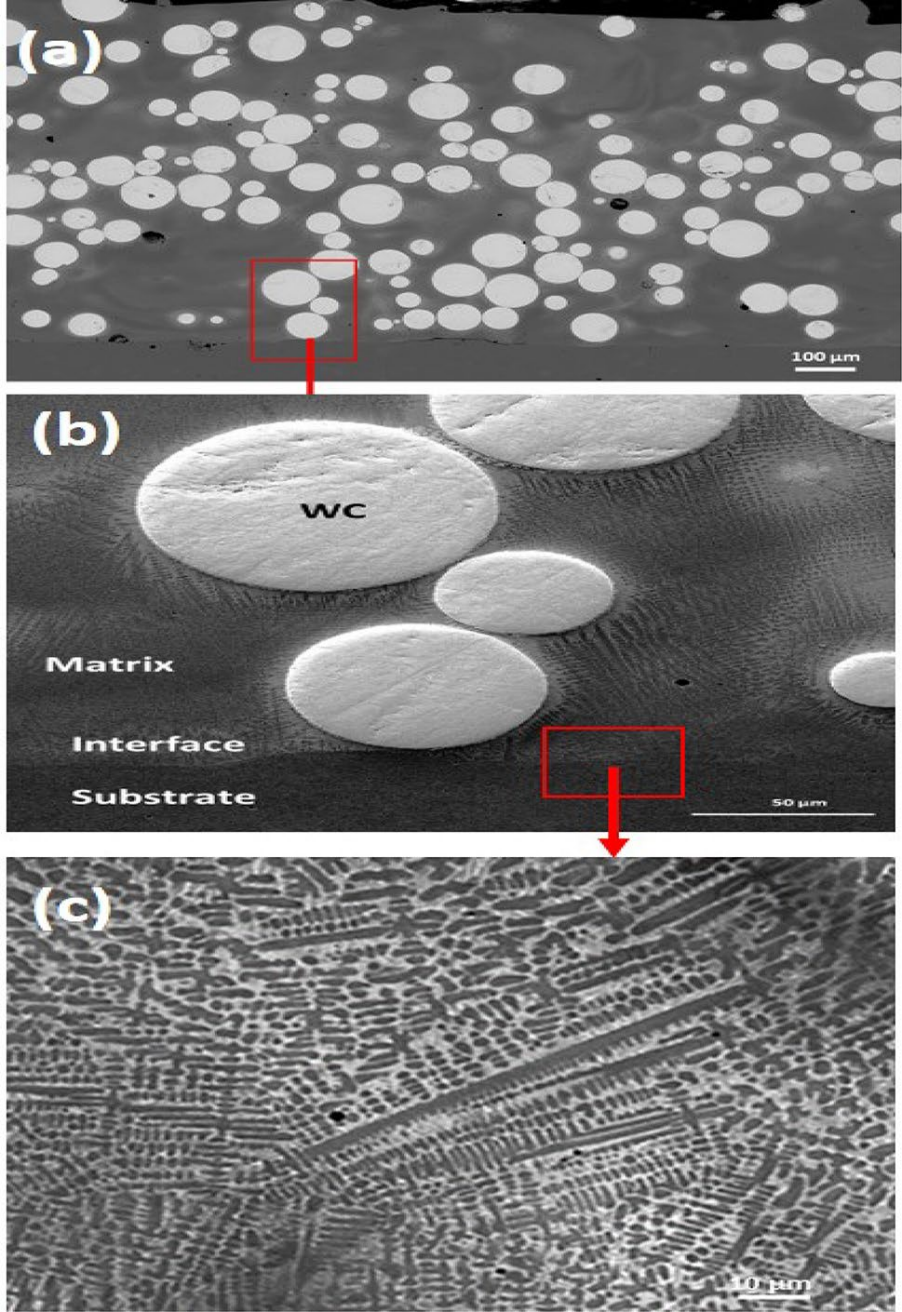
SEM micrographs of specimen produced at *F* = 90 g/min and *E* = 12 J/mm^2^: (**a**) deposited layer showing uniformly distributed spherical WC particles, (**b**) interface zone between deposit and substrate, and (**c**) higher-magnification view of the epitaxial dendritic structure within the interface zone (region indicated by red box in (**b**)).

The microstructure of specimens produced at the optimum condition (*F* = 90 g/min, *E* = 12 J/$mm^2$) is shown in Fig. 7a–c. The ANOVA results indicated a balanced contribution of *v* and *F* at these conditions, which is reflected in the high degree of carbide retention and matrix stability observed in the micrographs. The microstructure consists of a dense composite with excellent interfacial bonding between the WC particles and the matrix. Moreover, the spherical WC particles are uniformly distributed within the matrix and retain their original spherical morphology, as shown in Fig. 7a. This morphology is essential for maximizing wear resistance. The homogeneous distribution of the WC particles in the matrix is attributable to the low *E* used, which increases melt pool viscosity and minimizes the time available for WC particles to sink to the bottom of the melt pool. This observation is in good agreement with results reported in^6,18^. This uniform distribution further indicates that minimal dilution was achieved. Fig. 7b shows the interface zone, which consists mainly of epitaxial dendritic structures and occasionally contains a few WC particles. The dendritic grains are fine and uniformly distributed throughout the entire interface zone, as shown in Fig. 7c.

**Fig. 8:**
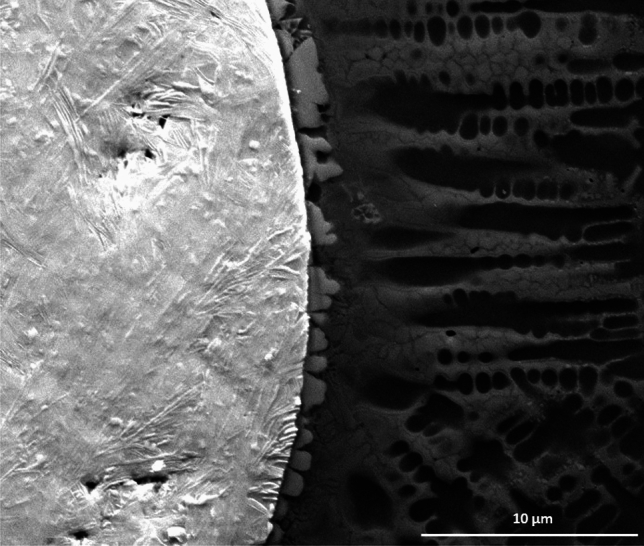
SEM of the interface of WC particle.

However, higher magnification images reveal partial dissolution at the carbide matrix interface, as shown in Fig. 8, while the WC particles themselves are mostly preserved. This limited dissolution enhances interfacial bonding with the matrix and contributes to improved matrix hardness^6,7,14,18^. Some of the investigated specimens exhibited minor porosity and microcracks that were negligible. To quantitatively evaluate the internal integrity of the tracks, digital image thresholding analysis was performed on representative cross sections. The specimens produced under the optimized processing window exhibited an exceptionally low average porosity area fraction of 0.38% (well below 0.5%), consisting primarily of isolated, sub-micron spherical gas pores, quantitatively confirming the near defect free status of the deposits. This high density and structural soundness is structurally attributed to the unique combination of low *E*, short *T*, and high *F*. These conditions feature lower values for *E* and *T* than those reported in previous studies, while the *F* is significantly higher. Consequently, the low *E* and short *T* regime effectively prevented excessive melting and macroscopic dissolution of carbides. In addition, at high *F*, the dense powder stream exerted a pronounced shielding effect, absorbing a fraction of the incident laser beam and preventing it from directly overheating the melt pool and substrate. As a result, the peak melt pool temperature decreased, leading to fewer melted WC particles, reduced formation of brittle intermetallic phases, and the attainment of robust deposit thicknesses. The attainment of these highly refined, near-defect free layers is heavily dictated by the underlying solidification physics governing high rate directed energy deposition. As recently demonstrated by Hodgir et al. ^25^, aggressively increasing processing boundaries to enhance build productivity fundamentally alters the local thermal history, where slower cooling rates can encourage coarse columnar structures, localized solute segregation, and gas pore entrapment. In the present study, this rapid deposition trade-off is precisely controlled by operating within a low energy density and short interaction time window ($T = 0.036$ s). This optimal parameter combination re-establishes steep thermal gradients (*G*) and swift cooling rates ($G \times R$), which effectively bypasses the typical coarsening mechanisms seen in rapid builds. This constricted thermal window plays a vital dual role: it strictly limits the macro-dissolution kinetics at the carbide-matrix interface to prevent brittle phase networks, while simultaneously driving dendritic refinement and solid solution strengthening to yield a peak matrix hardness of $550 \pm 15$ HV.

### Hardness measurements

**Fig. 9:**
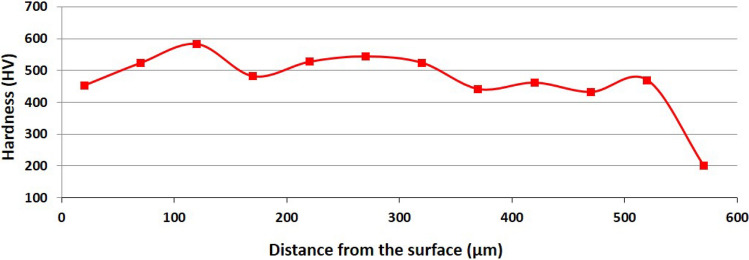
Hardness of specimen made at *E* of 12 J/mm^2^ and *F* of 90 g/min.

According to the powder specification, the hardness of the Fe-based alloy powder used in this study is 390 HV. Hardness measurements performed on the WC particles yielded values of 2370 HV. Therefore, to precisely evaluate the hardness of the deposited layer while excluding the contribution of the WC particles, all matrix hardness measurements were conducted in WC-free regions of the cross section. The values obtained accurately reflect the strengthening mechanisms within the matrix. The matrix hardness reached 550 ± 15 HV, which is in good agreement with the value reported for the matrix in^14^ and indicates limited dissolution of the WC particles. Matrix hardness measured at the optimum condition (550 ± 15 HV) was the highest among all tested parameter sets, confirming that the combination of *E* = 12 J/$mm^2$ and *F* = 90 g/min simultaneously maximises both productivity and mechanical performance. Fig. 9 shows the hardness profile of the specimen produced at the optimum condition (*E* = 12 J/$mm^2$, *F* = 90 g/min). The hardness values increase from the deposit substrate interface toward the top of the layer. This gradient is attributed to the higher cooling rate near the top surface, which produces a finer microstructure. Moreover, during solidification the WC particles act as heterogeneous nucleation sites, refining the matrix grains. In addition, the partially dissolved WC induces in-situ carbide precipitation, providing second phase strengthening. The lower hardness values near the bottom of the deposit are related to dilution from the substrate. The overall hardness trend agrees well with values reported in^6,7,10,14,17,18^, although the absolute values differ because of variations in processing parameters.

### Residual stress and cracking susceptibility analysis

The deposition of metal matrix composites with high ceramic reinforcement fractions ($50~\text {wt\% WC}$) introduces severe susceptibility to residual tensile stress accumulation and brittle macro-cracking. This behavior is primarily governed by the significant mismatch between the coefficients of thermal expansion (CTE) of the spherical WC reinforcement particles ($\alpha _{\text {WC}} \approx 5 \times 10^{-6}~\text {K}^{-1}$) and the Fe-based self-fluxing matrix alloy ($\alpha _{\text {Fe}} \approx 12 \times 10^{-6}~\text {K}^{-1}$). During the rapid solidification and cooling cycles inherent to the LMD process, this CTE differential generates substantial localized tensile stresses concentrated at the particle–matrix interfaces. Under conventional high heat inputs, this stress profile is heavily exacerbated by steep thermal gradients (*G*) and extensive interfacial dissolution of WC. This dissolution overly enriches the molten pool with W and C atoms, promoting the formation of continuous, brittle eutectic carbide networks that act as preferential pathways for macro-crack propagation. Crucially, despite the complete absence of substrate preheating in the current experiments, macro-cracking was entirely suppressed within the optimized operating window ($E = 12~\text {J/mm}^2$, $F = 90~\text {g/min}$). This structural integrity is directly attributable to the combined effects of a low energy density and a high powder mass flow rate. The dense, coaxial powder stream forms a highly concentrated spatial particle cloud directly beneath the nozzle, which exerts a pronounced optical and thermal shielding effect. By attenuating the peak laser energy reaching the melt pool, this shielding action lowers the maximum temperature attained by the molten pool, thereby significantly flattening the thermal gradient (*G*) and reducing the magnitude of residual transient stresses. Furthermore, the limited thermal exposure curtails the macro-dissolution kinetics of the WC particles, preserving the soft, ductile primary matrix phase and arresting microcrack initiation at the interface. This dual thermal-buffering mechanism effectively manages the mechanical reliability of thick composite layers, offering a highly robust and economical path for defect-free industrial remanufacturing without complex external hardware or preheating setups.

### Strategic relevance for governance and industry

To establish the true industrial relevance of high-rate LMD remanufacturing, the process must be evaluated using quantitative material deposit economy and energetic sustainability metrics derived directly from the experimental process data. As plotted across the parameter matrix, the volumetric productivity potential (build rate, *B*) reaches a maximum threshold of $4083.4~\text {mm}^3/\text {min}$ at the optimized parameters ($E = 12~\text {J/mm}^2$, $F = 90~\text {g/min}$), and extends up to $5959.4~\text {mm}^3/\text {min}$ under the maximum high-productivity load ($F = 140~\text {g/min}$). From a circular manufacturing perspective, material utilization efficiency is strictly governed by the Powder Melting Efficiency (PME). Operating within the identified stable processing window ($E \ge 12~\text {J/mm}^2$) ensures that the PME remains securely stabilized above 42% (peaking at 46.6% for the optimized run), which directly minimizes raw powder overspray waste. This represents a massive reduction in material consumption compared to the sub-optimal low-energy boundaries ($E = 7.5~\text {J/mm}^2$), where the severe laser-beam attenuation caused by the dense spatial particle cloud drives a complete collapse of the PME to a wasteful 5.3%–5.7%. By preventing this catchment failure, the optimized window slashes powder raw material waste by more than 85%, ensuring a highly sustainable material balance. Furthermore, process energy optimization can be quantified via the Specific Volumetric Melting Efficiency ($m_\mu$, expressed in $\text {mm}^3/\text {J}$), which dictates the volume of fully dense Fe-WC composite successfully deposited per Joule of delivered laser energy. The experimental data shows that operating at a low laser energy density of $12~\text {J/mm}^2$ combined with a heavy powder mass load maximizes $m_\mu$ to its absolute peak recorded values of $1.97~\text {mm}^3/\text {J}$ and $1.98~\text {mm}^3/\text {J}$ for $F = 90~\text {g/min}$ and $F = 140~\text {g/min}$, respectively. This highly efficient conversion rate demonstrates that the powder-induced shielding stream acts as an optimized thermal buffer. By concentrating energy absorption directly within the dense moving powder cloud rather than wasting excessive thermal energy via conduction deep into a heavily diluted steel substrate, the net embodied carbon and energy footprint per unit volume of repaired component is minimized. These measurable parameters provide industrial policymakers with a rigorous, scalable baseline to validate laser metal deposition as an energy-efficient tool for heavy-industry component life extension.

## Conclusion

The present study demonstrates the laser metal deposition process using an Fe-based self-fluxing alloy reinforced with 50 wt% WC, offering a high deposition rate solution for repairing and enhancing engineering components with superior performance and economic benefits. The specific conclusions are summarized below.

1. Powder feed rate (*F*) is the dominant factor (57–90 $\%$ contribution) governing dilution and aspect ratio. High feed rates (>90 g/min) consistently produce dilution <10$\%$ via laser-beam shielding, thereby preserving deposit chemistry and metallurgical bonding.
2. Scanning speed (*v*) exerts primary control (45–77 $\%$ contribution) over build rate and powder melting efficiency. The identified low energy density, short interaction time regime delivers balanced productivity without compromising layer integrity.
3. The combination of low energy density, short interaction time, and high powder feed rate yields near defect free deposits with excellent carbide retention, uniform WC distribution, and minimal interfacial dissolution.
4. At the optimized condition (*E* = 12 J/$mm^2$, *F* = 90 g/min), the matrix hardness reaches 550 HV through grain refinement and in-situ carbide precipitation, while large deposit thicknesses are attained owing to the shielding effect.

The demonstrated process stability under low thermal input and high mass flow provides a robust technical foundation for smart, low-carbon manufacturing transitions. It enables policymakers to accelerate Industry 5.0 adoption and support decarbonization targets through scalable, repair oriented LMD process

## Future work

- Future research should integrate in-situ melt pool monitoring (high speed imaging and infrared thermography) with machine learning based feedback control to enable real time parameter adaptation, thereby fully realizing Industry 5.0’s vision of intelligent and human-centric manufacturing.
- Service condition validation (wear, corrosion, and fatigue testing) should be performed on repaired full scale components to confirm remanufacturing performance.
- The optimized parameter set should be extended to other ceramic reinforced materials.
- Future experiments employing independent variation of spot size or dual laser configurations will be conducted to fully decouple the individual contributions of energy density and interaction time.

## Data Availability

The datasets used and/or analysed during the current study are available from the corresponding author on reasonable request
